# Therapeutic Applications of Physalins: Powerful Natural Weapons

**DOI:** 10.3389/fphar.2022.864714

**Published:** 2022-04-05

**Authors:** Cássio Santana Meira, José Waldson Capinan Soares, Bruna Padilha Zurita Claro dos Reis, Luciano Vasconcellos Pacheco, Ivanilson Pimenta Santos, Dahara Keyse Carvalho Silva, Julia Costa de Lacerda, Sérgio Ricardo Teixeira Daltro, Elisalva Teixeira Guimarães, Milena Botelho Pereira Soares

**Affiliations:** ^1^ SENAI Institute of Innovation in Health Advanced Systems (CIMATEC ISI SAS), University Center SENAI/CIMATEC, Salvador, Brazil; ^2^ Gonçalo Moniz Institute, Oswaldo Cruz Foundation (IGM-FIOCRUZ/BA), Salvador, Brazil; ^3^ Department of Life Sciences, State University of Bahia (UNEB), Salvador, Brazil; ^4^ Bahiana School of Medicine and Public Health, Bahiana Foundation for the Development of Sciences, Salvador, Brazil

**Keywords:** physalins, Physalis, pharmacological properties, Solanaceae, Withanolides

## Abstract

Physalins, or 16,24-cyclo-13,14-seco steroids, are compounds belonging to the class of withanolides that can be found in plants of Solanaceae family, mainly in species belonging to the genus *Physalis* spp., which are annual herbaceous plants widely distributed in tropical and subtropical regions of the world. Physalins are versatile molecules that act in several cell signaling pathways and activate different mechanisms of cell death or immunomodulation. A number of studies have shown a variety of actions of these compounds, including anticancer, anti-inflammatory, antiparasitic, antimicrobial, antinociceptive, and antiviral activities. Here we reviewed the main findings related to the anticancer, immunomodulatory, and antiparasitic activities of physalins and its mechanisms of action, highlighting the \challenges and future directions in the pharmacological application of physalins.

## Introduction

The use of medicinal plants for the treatment of diseases is a recognized practice and used for thousands of years by different civilizations around the world (Ahmad et al., 2006). Based on technological advances in chemistry for the isolation and identification of natural products, it has been possible to uncover the structure and biological potential of countless compounds from plants (Kartz and Baltz, 2016; Prieto-Martínez et al., 2019). These phytochemical compounds exhibit a highly rich biochemical complexity and diversity, comprising molecular structures unique compared to other compounds artificially synthesized. Thus, natural products are recognized as a promising source for the prospect of therapeutic agents (Dias et al., 2012; Newman and Cragg, 2020).

**FIGURE 1:**
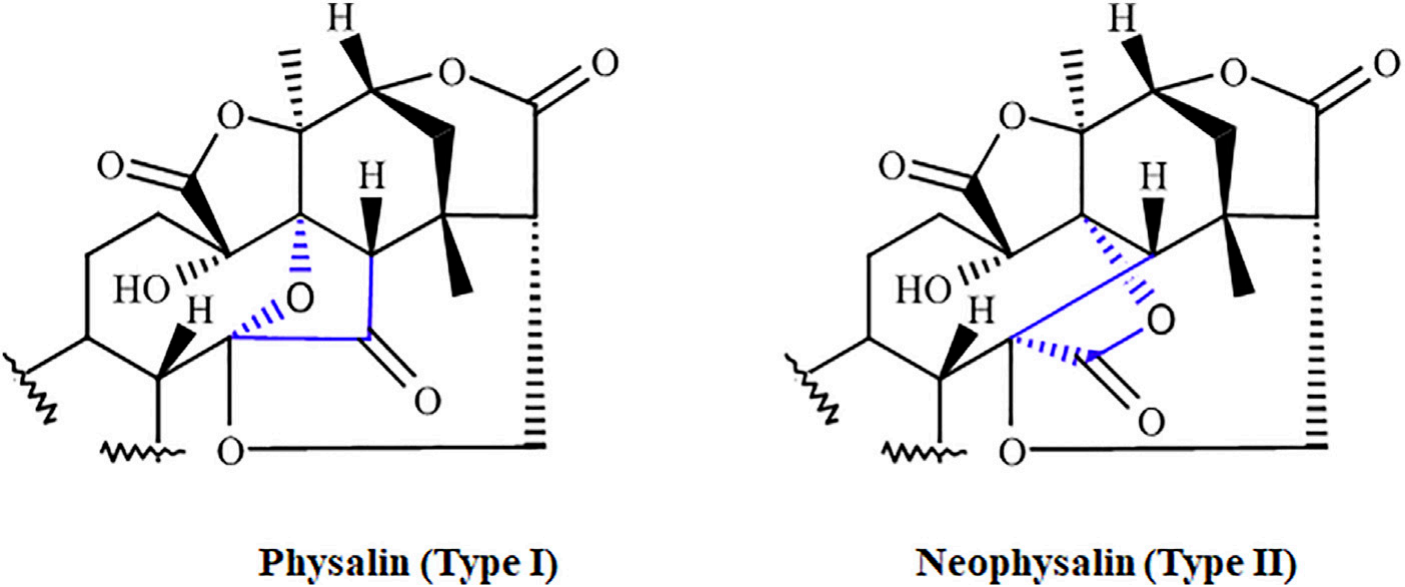
The two subclasses of physalins. Physalins (Type I), in which C-14 is linked to C-17 through oxygen to form an acetal bridge, and neophysalins (Type II), in which C-14 is linked to C-16, while esterization of C-15/C-17 forms a lactone. The main differences between the two types are highlighted in blue.

**FIGURE 2:**
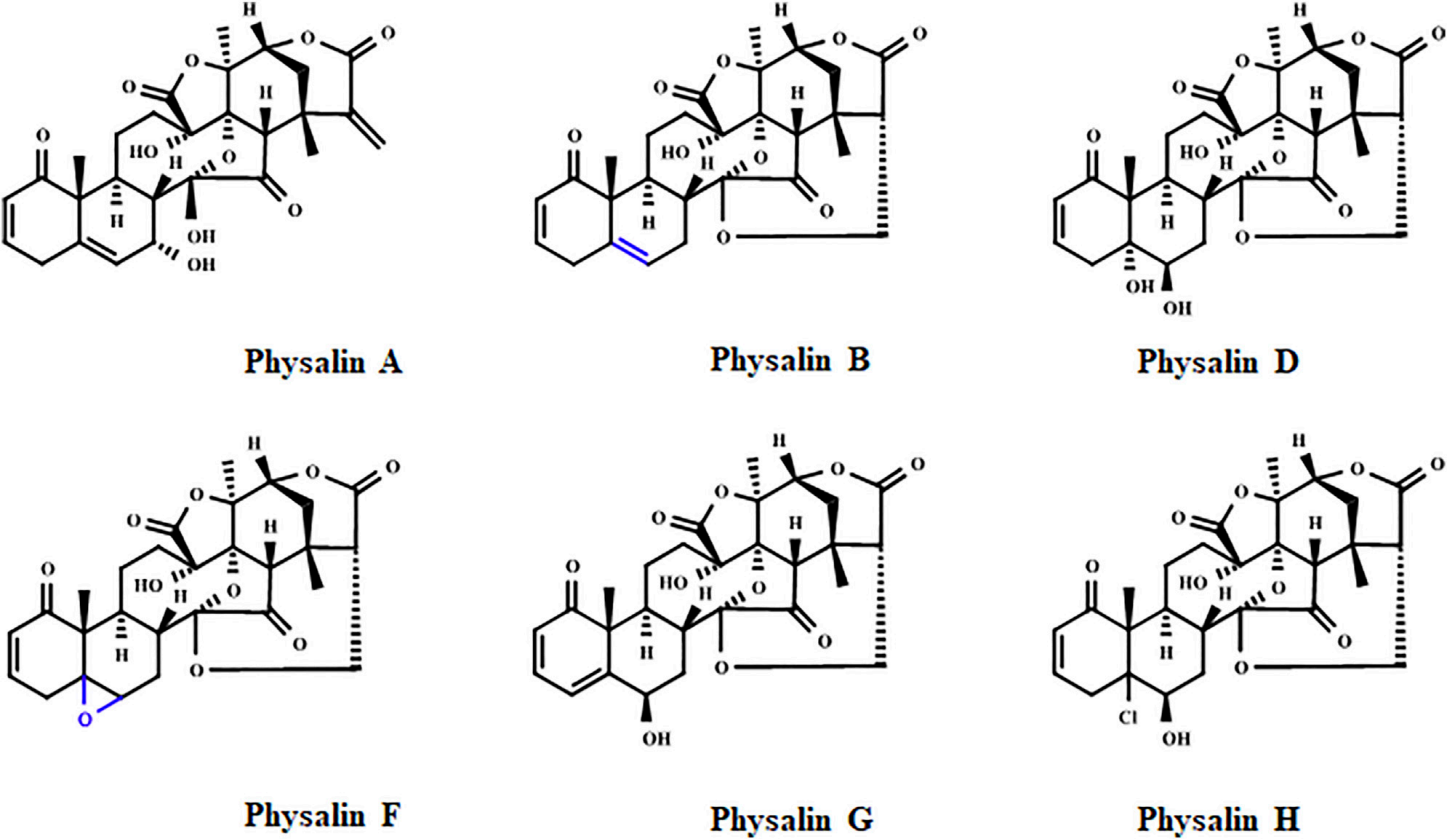
Chemical structure of the physalins A, B, D, F, G, and H. The epoxy group of physalin F and the double bond for physalin B, which may contribute to the potent cytotoxic effect of these physalins, are highlighted in blue.

Physalins, or 16,24-cyclo-13,14-seco steroids, are withanolides compounds which exhibit several promising pharmacological properties (Tomassini et al., 2000; Zhang and Ong, 2016; Sun et al., 2017a). Physalins are found in plants belonging to the Solanaceae family, mainly in species of the genus *Physalis* spp., which are annual herbaceous plants widely distributed in tropical and subtropical regions of the world, and are known for their therapeutic and curative properties (Li et al., 2018). In 1969, when the first physalin was isolated (physalin A) from *Physalis alkekengi* var. franchetii, the studies about the biological activities of this class of molecules began (Matsuura et al., 1970). In general, physalins are classified into two subclasses, physalins (Type 1), in which C-14 is linked to C-17 through oxygen to form an acetal bridge, and neophysalins (Type II), in which C-14 is linked to C-16, while esterization of C-15/C-17 forms a lactone (Figure 1). At the time of writing, the chemical structures of more than 75 different physalins have been described (reviewed by Wu et al., 2021), being physalins A, B, D, F, G, and H the most extensively studied (Figure 2).

Moreover, several studies have shown diverse biological activities of these compounds, including anticancer, anti-inflammatory, antimicrobial, antinociceptive, antiparasitic, and antiviral (Soares et al., 2003; Meira et al., 2013; Lima et al., 2014; Wang et al., 2018; reviewed by; Wu et al., 2021). In this context, this review aims to describe the main findings and mechanisms of action related to the anticancer, immunomodulatory and antiparasitic effects of the physalins class.

## Immunomodulatory Activity

Physalins are pleiotropic molecules capable of interacting with various components involved in the onset and resolution of inflammation (Soares et al., 2003; Jacobo-Herrera et al., 2006; Ding et al., 2019; Lin et al., 2020). These interactions allow several physalins to act as potent anti-inflammatory and immunosuppressive agents, as shown in different *in vitro* and *in vivo* systems (Tables 1, 2).

**TABLE 1:** *In vitro* immunomodulatory activity of physalins.

References	Physalins	Main results
Soares et al. (2003)	B, D, F, and G	Physalins B, F, or G, but not D, inhibited NO production by macrophages. In addition, physalin B inhibited TNF, IL-6, and IL-12 production by macrophages
Jacobo-Herrera et al. (2006)	B, D, and F	Physalins B and F, but not D inhibited NFκB activation
Soares et al. (2006)	B, D, F, and G	Physalins B, F or G, but not D, inhibited lymphroproliferation induced by Con A. In addition, physalin B inhibited lymphroproliferation in the mixed lymphocyte culture reaction and IL-2 production
Brustolim et al. (2010)	F	Physalin F did not promote the translocation of the glucocorticoid receptor from the cytoplasm to the nucleus
Yu et al. (2010)	H	Inhibition of lymphroproliferation induced by con A and by the mixed lymphocyte culture reaction. Also, a decrease of IL-2, IFNy, and increase of IL-4, IL-10, and HO-1 production
Ji et al. (2012)	A, G, L, O, and isophysalin A	Inhibition LPS-induced NO production by macrophages
Pinto et al. (2010)	F	Inhibition of lymphroproliferation of PBMC in HAM/TSP subjects and reduction of the levels of IL-2, IL-6, IL-10, TNF, and IFN-γ, but not IL-17A, in supernatants of PBMC cultures
Sun et al. (2017a)	V, VI, VII, VIII, and IX	Inhibition LPS-induced NO production by macrophages
Sun et al. (2017b)	X and aromaphysalin B	Inhibition LPS-induced NO production by macrophages
Yang et al. (2017)	E	Inhibition of TNF and IL-6 expression and secretion and NF-κβ nuclear translocation on macrophages cultures
Ding et al. (2019)	D	Regulation of macrophage M1/M2 polarization via the STAT1/6 pathway
Qiu et al. (2008)	G, I, W, X, Y, Z, and II	Inhibition LPS-induced NO production by macrophages
Lin et al. (2020)	A	Inhibition of PGE_2_, NO, IL-1β, IL-6, and TNF in LPS-induced RAW 264.7 cells and suppression of JNK/AP-1 and IκB/NF-κB signaling pathways
Zhang et al. (2020)	B	Reduction of the levels of TNF, IL-6, and IL-1β on LPS-stimulated RAW 264.7 cells
Wang et al. (2021a)	A	Reduction of the release of NO, PGE_2_ and TNF by blocking the activation of NF-κB signaling pathway

AP-1, activator protein-1; Con A, concanavalin A; HAM/TSP, Human T-Cell Lymphotropic Virus Type 1-Associated Myelopathy/Tropical Spastic Paraparesis; HO-1, heme oxygenase-1; IFN- γ, Interferon gamma; IκB, Ikappa B kinase; IL-1β, Interleukin-1 beta; IL-2, Interleukin-2; IL-6, Interleukin-6; IL-10, Interleukin-10; IL-12, Interleukin-12; IL-17A, Interleukin-17A; JNK; c-Jun N-terminal kinase; LPS, lipopolysaccharide; NF-κB, Nuclear factor kappa-light-chain-enhancer of activated B cells; NO, nitric oxide; STAT1, Signal transducer and activator of transcription 1; STAT6, Signal transducer and activator of transcription 6; PBMC, *peripheral blood mononuclear cell*; PGE2, prostaglandin E2; TNF, tumor necrosis factor.

**TABLE 2:** *In vivo* immunomodulatory activity of physalins.

References	Physalins	Route/dose	Model	Main results
Soares et al. (2003)	B, F, and G	I.P./0.5 or 1 mg/kg	Endotoxic shock-induced by LPS in BALB/c mice	Physalins, especially physalin B, protected mice against a lethal lipopolysaccharide challenge
Vieira et al. (2005)	B and F	S.C./2–20 mg/kg	Intestinal ischaemia in C57BL/6 mice and reperfusion injury	Physalins prevented neutrophil influx and the increase in vascular permeability in the intestine and lungs. In addition, inhibited TNF production and increased IL-10 levels
Soares et al. (2006)	B, F, and G	Oral/1 mg/kg	Allogeneic transplant rejection model in BALB/c mice	Physalin B, F, or G prevented the rejection of allogeneic heterotopic heart transplant
Brustolim et al. (2010)	F	Oral/20 mg/kg	Collagen-induced arthritis model in DBA/1 mice	Decreased in paw edema and joint inflammation
Brustolim et al. (2010)	F	Oral/60 mg/kg	Allergic airway inflammation-induced by ovalbumin in BALB/c mice	Non effect in allergic airway inflammation
Pinto et al. (2010)	E	T.A./0.125, 0.25, and 5 mg/ear	TPA and oxazolone-induced dermatitis in Swiss mice	Reduction of ear edema/thickness, TNF, IFN-ɣ, NF-κB, and MPO activity
Yu et al. (2010)	H	I.P./4.4, 8.8, and 17.6 mg/kg	DNFB-induced delayed type hypersensitivity reaction in BALB/c mice	Physalin H dose-dependently suppressed CD4^+^ T cell mediated delayed-type hypersensitivity reactions and suppressed antigen-specific T-cell response in OVA immunized mice
Lin et al. (2020)	A	I.P./2.5, 5 or 10 mg/kg	Carrageenan-induced paw edema in ICR mice	Reduction of paw edema accompanied by NO, MDA, and TNF decrease. In addition, antioxidant factor levels (SOD, CAT and GPx) were all increased by the treated with physalin A
Zhang et al. (2020)	B	I.P./10 or 20 mg/kg	Acute colitis-induced by DSS in BALB/c mice	Treatment with physalin B ameliorated clinical features of ulcerative colitis through modulation of NF-κB pathway and related pathways
Wang et al. (2021b)	A	Gastric perfusion/5, 10, and 20 mg/kg	Carrageenan-induced paw edema in SD rats and acetic acid-induced capillary permeability in KM mice	Reduction paw edema and the vascular permeability in a dose-dependent manner

CAT, catalase; CD4; cluster of differentiation 4; DNFB, 2,4-dinitrofluorobenzene; DSS, dextran sulfate sodium; GPx, glutathione peroxidase; IFN-γ, Interferon gamma; IL-10, Interleukin-10; I.P., intraperitoneal route; MDA, malondialdehyde; MPO, Myeloperoxidase; NFκB, Nuclear factor kappa-light-chain-enhancer of activated B cells; OVA; ovalbumin; S.C., subcutaneous; SOD, Superoxide dismutase; TNF, tumor necrosis factor T.A., topical application; TPA, 12-O-tetradecanoyl-phorbol-13-acetate.

**FIGURE 3:**
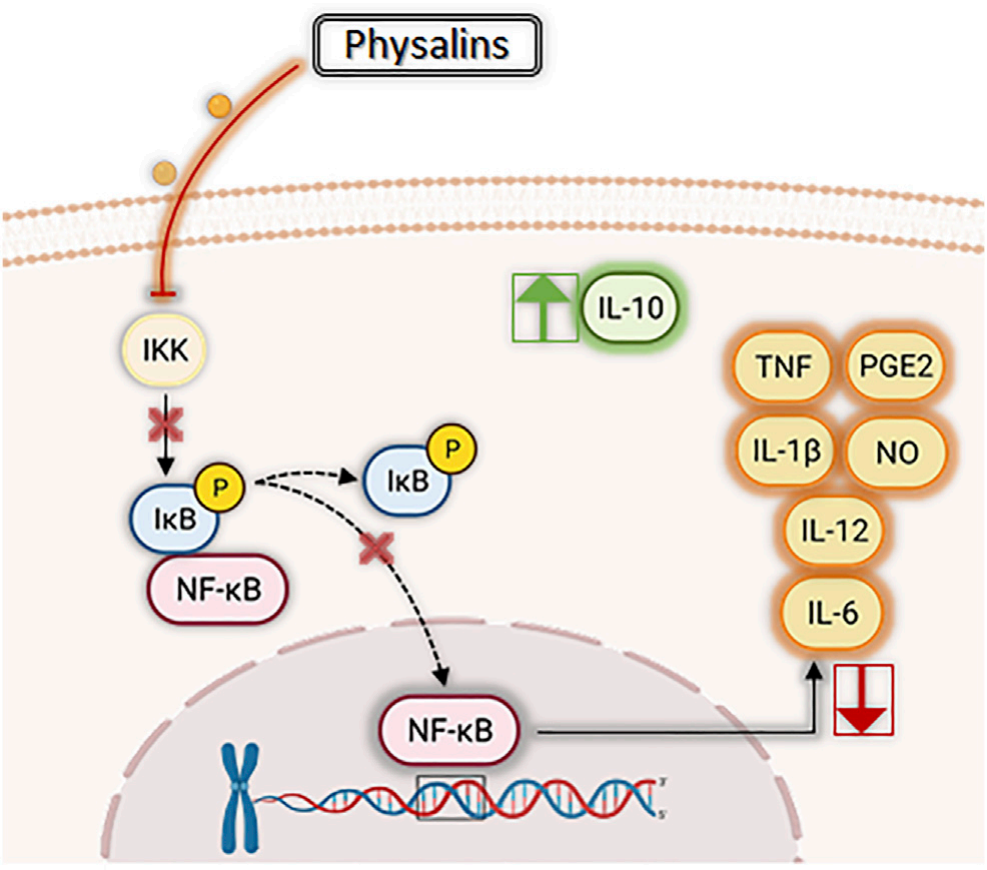
Main anti-inflammatory effects of physalins. In general, physalins suppress phosphorylation of iκB proteins and impair NF-κB translocation to the nucleus. NF-κB is involved in the regulation of several pro-inflammatory genes, and thus suppression of its activity by physalins results in inhibition of pro-inflammatory mediators, such as interleukins (IL)-1β, IL-6, IL-12, nitric oxide (NO), prostaglandin E_2_ (PGE_2_), and tumor necrosis factor (TNF). In addition, some physalins (such as physalin F and H) increase the production of IL-10, a well-known anti-inflammatory cytokine.

Several *in vitro* studies (Table 1) demonstrate that physalins can inhibit the production of nitric oxide (NO) in macrophages cultures stimulated with lipopolysaccharide (LPS) and/or interferon gamma (IFN-ɣ) (Soares et al., 2003; Qiu et al., 2008; Ji et al., 2012; Sun et al., 2017b; Lin et al., 2020; Wang et al., 2021a). NO is produced from L-arginine by the action of the enzyme nitric oxide synthase (NOS), playing an important role in inflammatory responses (Förstermann and Sessa, 2012). Furthermore, physalins A, B, E, F, and G can inhibit the production of several inflammatory molecules, such as interleukin (IL)-1β, IL-6, IL-12, prostaglandin E_2_ (PGE_2_), and tumor necrosis factor (TNF), by activated macrophages (Figure 3) (Soares et al., 2003; Yang et al., 2017; Lin et al., 2020; Zhang et al., 2020; Wang et al., 2021b). Most of these effects are attributed to the inhibition of nuclear factor kappa-light-chain-enhancer of activated B cells (NF-κB), a transcription factor involved in the regulation of several pro-inflammatory genes (Figure 3) (Jacobo-Herrera et al., 2006; Yang et al., 2017; Lin et al., 2020; Zhang et al., 2020).

Moreover, physalin D was shown to promote polarization of macrophages with a M1 to a M2 profile, possibly via the signal transducer and activator of transcription (STAT)-1/6 pathway (Ding et al., 2019). Macrophages with a M1 phenotype are characterized by production of NOS and pro-inflammatory cytokines, such as IL-1β and TNF, being involved in the progress of inflammatory response. On the other hand, macrophages with a M2 phenotype are characterized by production of arginase 1 and IL-10, being associated with resolution of inflammation and tissue repair (Wang et al., 2021a). In this sense, the polarization towards a M2 phenotype promoted by physalin D is attractive for applications in the treatment of inflammatory diseases (Ding et al., 2019).

The immunosuppressive potential of physalins B, D, F, G, and H were also investigated (Soares et al., 2006; Yu et al., 2010; Pinto et al., 2010). With the exception of physalin D, physalins B, F, G, and H (at concentrations below 5 µg/ml) showed a potent antiproliferative effect in concanavalin A-stimulated lymphocytes or in mixed lymphocyte reaction assays (Soares et al., 2006; Yu et al., 2010). The inhibition of lymphocyte proliferation, promoted by physalin F, was induced by a cell cycle arrest in the G1 phase (Yu et al., 2010). Pinto et al. (2010) demonstrating that physalin F induced apoptotic cell death of lymphocytes from patients with human T-lymphotropic virus type 1 (HTLV-1) (Table 1).

The suppression of lymphocyte proliferation induced by different physalins is accompanied by a reduction in cytokines related to clonal lymphocyte activation and expansion, such as IL-2 and IFN-γ (Soares et al., 2006; Yu et al., 2010; Pinto et al., 2010). Yu et al. (2010) also demonstrated that physalin H modulates the Th1/Th2 balance, by decreasing the secretion of Th1-associated cytokines (IL-2 and IFN-γ) and increasing the secretion of Th2-associated cytokines (IL-4 and IL-10), thus reversing Th1 polarization *in vitro*. The subsets of T-helper cells are useful for classifying the immune responses that occur in the elimination of microbial pathogens (Hirahara and Nakayama, 2016). Th1 responses are associated with cell-mediated immune responses and phagocyte-dependent protective responses, whereas Th2 responses are related to host defense against multi-cellular parasites and allergies and atopic illnesses (Raphael et al., 2015). Interestingly, physalin H induced heme oxygenase-1 protein expression in mouse T lymphocytes, a response which is associated with a protective effect against autoimmune diseases (Chauveau et al., 2005).

The anti-inflammatory effects of physalins have also been validated in various animal models (Table 2). The initial work by Soares et al. (2003) demonstrated the anti-inflammatory action of physalins B, F, and G (at 0.5 or 1 mg/kg) in a mouse model of endotoxic shock, protecting mice against a lethal dose of LPS and decreasing the production of the pro-inflammatory cytokine TNF (Soares et al., 2003). Vieira et al. (2005) showed that physalins B and F (in 20, 2, or 0.2 mg/kg) reduced vascular permeability, decreased serum TNF concentrations and increased the production of IL-10 in a model of intestinal injury by ischemia and reperfusion in mice.

Moreover, physalin E, applied topically, (0.125, 0.25 and 0.5 mg/per ear, 20 µl) revealed anti-inflammatory effects in both acute and chronic models of 12-O-tetradecanoyl-phorbol-13-acetate-induced dermatitis (TPA) and oxazolone, respectively (Pinto et al., 2010). Through immunohistochemical analysis, a reduction of TNF and NF-κB was observed in the ears of mice treated with physalin E (0.5 mg/kg), indicating an involvement of NF-κB pathway in its mechanism of action (Pinto et al., 2010). In agreement with this data, physalin B, when tested in a mouse model of acute colitis-induced by dextran sulfate sodium (DSS), also suppressed the NF-κB cascade by reducing the p-NF-kB p65 and p-iκβα, leading to alleviation of the symptoms and pathological features of ulcerative colitis (Zhang et al., 2020).

Physalin A, when tested in a carrageenan-induced model, significantly reduced paw edema (Lin et al., 2020; Wang et al., 2021b). In the work of Lin et al. (2020), a reduction of paw edema was achieved through the reduction of NO, TNF, and malondialdehyde (MDA) and increase in the activity of antioxidant enzymes (catalase, superoxide dismutase, and glutathione peroxidase) (Table 2).

Since physalins have a steroidal chemical structure, their interaction with glucocorticoid receptors was investigated as a possible mechanism of action. Most investigations were conducted using mifepristone (or RU-486), which is a steroidal antiprogesterone that works as an antagonist of glucocorticoid receptors. Pretreatment *in vivo* with mifepristone (25 mg/kg) reversed the anti-inflammatory effects of physalins B and F in a model of intestinal injury by ischemia and reperfusion in mice and the anti-inflammatory effects of physalin E in TPA-induced dermatitis (Vieira et al., 2005; Pinto et al., 2010). However, these data were not supported by *in vitro* experiments with macrophage cultures, which demonstrated, that in the presence of mifepristone, the anti-inflammatory effects of physalins B and E, were not reduced, suggesting that these molecules do not depend on glucocorticoid receptors to exert their anti-inflammatory effects (Soares et al., 2003; Yang et al., 2017). Additionally, the hypothesis that the action of physalins is independent of a binding with glucocorticoid receptors is experimentally supported by the fact that treatment with physalin F does not promote the translocation of the glucocorticoid receptor from the cytoplasm to the nucleus (Brustolim et al., 2010).

Lastly, physalins B, F, G, and H also demonstrated their immunosuppressive effect in experimental animal models of immune-mediated diseases (Table 2). Physalins B, F, and G, when evaluated in a murine model of allogeneic transplantation, inhibited graft absorption and the local inflammatory response (Soares et al., 2006). In addition, when evaluated in a murine model of delayed-type hypersensitivity, physalin H reduced dose-dependently the edema in the animals’ ear and the proliferation of ovalbumin-specific T lymphocytes (Yu et al., 2010). Moreover, physalin F also reduced paw edema in a mouse model of collagen-induced arthritis. In contrast, physalin F did not ameliorate lung inflammation in a mouse model of allergic airway inflammation induced by ovalbumin, a Th2 associated disease (Brustolim et al., 2010).

## Antiparasitic Activity

Regarding the antiparasitic activities, many studies have investigated the leishmanicidal activity of physalins. Several physalins were shown to inhibit the proliferation of promastigote forms of *Leishmania* species of the New and Old Worlds, such as *L. amazonensis*, *L. braziliensis*, *L. chagasi*, and *L. major* (Table 3) (Choudhary et al., 2005; Choudhary et al., 2006; Choudhary et al., 2007; Guimarães et al., 2010). Among the physalins evaluated, physalin F stands out for having an inhibitory concentration of 50% (IC_50_) value of 1.4 µM against *L. amazonensis*, being more active than the other physalins tested and having an activity close that of amphotericin B (IC_50_ = 3.0 µM), a standard leishmanicidal drug (Guimarães et al., 2010).

**TABLE 3:** Antiparasitic activity of physalins.

Reference	Physalins	Main result
Choudhary et al. (2005)	1-3*, H, isophysalin B, and 5β,6β-epoxyphysalin B	All tested physalins showed leishmanicidal activity against promastigotes forms of *L. major* with IC_50_ values ranging from 0.9 to 38.9 µg/ml
Choudhary et al. (2006)	Isophysalin B, H, 6,7-dehydrophysalin H and 6,7-dehydrophysalin H	All tested physalins showed leishmanicidal activity against promastigotes forms of *L. major* with IC_50_ values ranging from 6.0 to 13.8 µM
Garcia et al. (2006)	B	Treatment, of *Rhodinius prolixus inoculated with T. rangeli*, with physalin B caused a reduction in hemocyte microaggregation and nitric oxide production and enhanced the parasitemia in the hemolymph
Choudhary et al. (2007)	1-2*	Both physalins showed leishmanicidal activity against promastigotes forms of *L. major* with IC_50_ values of 4.86 and 3.65 µg/ml
Guimarães et al. (2009)	B, D, F, and G	Physalins B and F, but not physalins D and G, inhibited amastigote development in macrophages cultures infected with *L. amazonensis*. In addition, physalin F reduced the lesion size, the parasite load and histopathological alterations in BALB/c mice infected with *L. amazonensis*
Guimarães et al. (2010)	B, D, F, and G	All tested physalins showed leishmanicidal activity against different species of *Leishmania*, in particular physalin F, which presented an IC_50_ value of 1.4 µM against promastigote forms of *L. amazonensis*
Sá et al. (2011)	B, D, F, and G	All tested physalins showed antimalarial activity against *Plasmodium falciparum* with IC_50_ values ranging from 2.2 to 55 µM. In addition, physalin D decreased parasitemia and mortality of *P. berghei*-infected mice
Castro et al. (2012)	B	Treatment with physalin B decreases the *T. cruzi* transmission by inhibiting epimastigote development in the insect vector *R. prolixus*
Meira et al. (2013)	B, D, F, and G	Physalins B and F showed trypanocidal activity against all forms of *T. cruzi*, inducing autophagic process, which ultimately may lead to necrotic death of the parasite

IC_50_, inhibitory concentration of 50%; *L. amazonensis*, *Leishmania amazonensis*; *L. major*, *Leishmania major*; *P. berghei*, *Plasmodium berghei*; *T. cruzi*, *Trypanosoma cruzi*; *T. rangeli*, *Trypanosoma rangeli*; 1-3*: Compounds: (1): 16,24-cyclo-13,14-secoergost-2-ene-18,26-dioic acid 14:17: 14:27-diepoxy-5α,6β,11β,13,20,22-hexahydroxy-1,15-dioxo-γ-lactone-δ-lactone, (2) 16,24-cyclo-13,14-secoergosta-18,26-dioic acid 5,6:14:17,14:27-triepoxy-13,20,22-trihydroxy-3α-methoxy-1,15-dioxo-γ-lactone-δ-lactone and (3) 16,24-cyclo-13,14-secoergost-2-ene-18,26-dioic acid 14:17,14:27-diepoxy-5α,13,20,22-tetrahydroxy-1,15-dioxo-γ-lactone-δ-lactone. 1-2*: Compounds: (1):16,24-Cyclo-13,14-seco-ergosta-2-ene-18,26-dioic acid 14,17:14,27-diepoxy-11β,13,20,22-tetrahydroxy-5α-methoxy-1,15-dioxo-γ-lactone-δ-lactone and (2): 16,24-Cyclo-13,14-seco-ergosta-2-en-18,26-dioic acid 14,17:14,27-diepoxy-5α,11β,13,20,22- tetrahydroxy-1,6,15-trioxo-γ-lactone-δ-lactone.

Physalins B, D, and F were also tested in an *in vitro* model of macrophage infection with *L. amazonensis and L. major*. Physalins B and F, but not physalin D, significantly (*p <* 0.05) reduced the number of infected macrophages and amastigotes in cultures infected with *L. amazonensis* or *L. major* (Guimarães et al., 2009). Since physalin F showed the best leishmanicidal effect against infected macrophages, it was also tested on *in vivo* model of cutaneous leishmaniasis. Topical treatment with physalin F significantly reduced the lesion size and parasite load when compared with mice treated with vehicle. Pathological features typically of lesion progression, such as necrotic areas, parasitism, and inflammatory infiltrate, were less frequently in animals treated with physalin F compared to vehicle-treated group (Guimarães et al., 2009).

The antiparasitic effect of some physalins (B, D, F, and G) has also been evaluated against *Trypanosoma cruzi*, another kinetoplastid protozoa (Table 3) (Meira et al., 2013). Physalins B and F showed anti-*T. cruzi* activity in epimastigote and trypomastigote forms of *T. cruzi*, being more potent than benznidazole, a reference drug. Physalins B and F presented IC_50_ values of 5.3 and 5.8 μM, respectively, against epimastigote forms, and IC_50_ values of 0.68 and 0.84 μM, respectively, against trypomastigote forms. Under the same conditions, benznidazole presented IC_50_ values of 10.8 and 11.4 μM against epimastigote and trypomastigote forms, respectively. A significant trypanocidal effect of physalin B and F, but not D and G, was also observed in cultures of infected macrophages. Regarding the mechanism of action against *T. cruzi*, the ultrastructural analysis of trypomastigotes treated with physalin B showed features suggestive of autophagic process, which ultimately may lead to necrotic death of parasite (Meira et al., 2013).

Interestingly, physalin B, when tested in *T. cruzi-*infected *Rhodnius prolixus*, especially by oral route, reduced or zeroed the number of parasites in the insect’s intestine. This effect was related to the increase in microbiota levels and production of reactive nitrogen species (Castro et al., 2012). The effect of physalin B on the development of *Trypanosoma rangeli* in *R. prolixus* was also evaluated. Pre-treatment of *R. prolixius* with blood containing different concentrations of physalin B caused a reduction in hemocyte microaggregation and nitric oxide production and enhanced the parasitemia in the hemolymph of insects (Table 3) (Garcia et al., 2006). These contrasting effects highlight the multi-target nature of physalins and the importance of the microenvironment to explain its effects in different models.

Lastly, the antimalarial activity of physalins B, D, F, and G was reported. These four physalins showed antimalarial activity *in vitro* against *Plasmodium falciparum*, with IC_50_ values ranging from 2.2 to 55 µM (Table 3) (Sá et al., 2011). Despite having the best effect *in vitro*, physalin F increased the parasitemia levels when tested *in vivo* in a *Plasmodium berghei* mouse model, probably due to its well-known immunosuppressive effects. In contrast, physalin D, the only one of the four tested without immunosuppressive effects, reduced parasitemia levels and increased the survival rate of *P. berghei*-infected mice (Sá et al., 2011).

Taken together, the data demonstrate the antiparasitic potential of the physalin class, in particular for the treatment of leishmaniasis. However, further studies are needed to better elucidate its mechanisms of action against these parasites.

## Anticancer Activity

Cancer is one of the leading causes of death worldwide, and the need for new treatments stimulated the evaluation of cytotoxic activity of physalins, mainly in leukemic, breast, lung, and prostate cancer cell lines, as shown in Table 4. Physalins B and F showed potent cytotoxic activities in CORL23 cells (large cell lung carcinoma) and MCF-7 cells (human breast cancer) cells, with IC_50_ values in the range of 0.4–1.92 µM (Lee and Houghton, 2005). IC_50_ values for physalin B and F below to 2 µM were also observed in other cancer cells lines, such as 22Rv1 cells (human prostate cancer), 796-O cells (human kidney cancer), A-498 cells (human kidney cancer), ACHN cells (human kidney cancer), CEM cells (human leukemia), C4-2B cells (human prostate cancer), HT1080 cells (human fibrosarcoma), HeLa cells (human cervical cancer), HCT-116 (human colorectal cancer), HL-60 cells (human promyelocytic leukemia), HuCCA-1 cells (human cholangiocarcinoma), and MOLT-3 cells (T lymphoblastic leukemia) (Magalhães et al., 2010; Ma et al., 2015; Yang et al., 2016; Sun et al., 2017a; Boonsombat et al., 2020). The evaluation of the structure-activity relationship indicates that the epoxy group of physalin F and the double bond for physalin B is crucial for the potent cytotoxic activity displayed (Figure 2) (Lee and Houghton, 2005; Damu et al., 2007; Magalhães et al., 2010; Boonsombat et al., 2020).

**TABLE 4:** Cytotoxic activity of physalins.

Reference	Physalins	Cell lines	Main results
Fang et al. (2003)	B, F, and H	BC1; Lu1; Col2; KB; LNCap; SW626; SKNSH and M109	Physalins, especially physalin B, showed broad cytotoxic activity in most of cell lines tested.
Lee and Houghton (2005)	B and F	COR L23; MCF-7	Both physalins displayed cytotoxic activity against cancer cell lines (IC_50_ values below to 2 µM), being physalin F more active
Magalhães et al. (2010)	B and D	CEM; HL-60; K562; HCT-8; MCF-7; MDA-MB-435; MDA-MB-231; PC-3 and B16	Both compounds displayed considerable cytotoxicity against several cancer cell lines, showing IC_50_ values ranging from 0.58 to 15.18 µg/ml for physalin B, and 0.28–2.43 µg/ml for physalin D. In addition, antitumour activity in mice transplanted with Sarcoma 180 tumour was confirmed
Damu et al. (2007)	B, D, F, J, U, and W	DU-145; 1A9; HCT116; LNCAP; KB; A431; A549; HCT-8; PC-3 and ZR751	Physalins, especially physalin F (EC_50_ values in the range of 0.3–1.3 µg/ml), showed broad cytotoxic activity in most of the cell lines tested
Ausseil et al. (2007)	B and C	DLD-1	Both physalins inhibit ubiquitin-proteasome pathway with EC_50_ values of 3.8 µM for physalin B and 4.4 µM for physalin C
Hosoya et al. (2008)	B and F	PANC1	Both physalins inhibit Headhog/GLI-mediated transcription and presented IC_50_ values of 2.6 and 5.3 µM against PANC1 cells
Vandenberghe et al. (2008)	B	DLD-1	Physalin B decreased the viability of DLD-1 cells by inhibiting the ubiquitin/proteasome pathway associated with the inhibition of NF-κB induced by TNF and with the induction of the Noxa protein, leading to death by apoptosis
Han et al. (2011)	A and B	CWR22Rv1 and C4B2B	Both physalins inhibit the proliferation of C42B and CWR22Rv1 cells by inducing apoptosis from the activation of the MAP kinase, ERK 1/2 and JNK pathways. In addition, both molecules reduced androgen receptor and prostate-specific antigen expression
Hsu et al. (2012)	B	A375 and A2058	Physalin B exhibits cytotoxicity in melanoma cancer cell lines by inducing apoptosis via the NOXA, caspase-3, and mitochondria-mediated pathways
Wu et al. (2012)	F	A498; ACHN and UO-31	Physalin F inhibited cell viability in human renal cancer cells by inducing cell apoptosis through the ROS-mediated mitochondrial pathway and suppressed NF-κB activation
He et al. (2013a)	A	A375-S2; HT1080; HepG2; HeLa; A549; U937; HCT116; A431; MCF-7 and HL-60	Physalin A showed broad cytotoxic activities towards most of the cell lines tested. In HT1080 cells, induced apoptosis associated with caspase-3 and caspase-8 activation and also induced autophagy
He et al. (2013b)	A	A375-S2	Physalin A induced apoptotic cell death via p53-Noxa-mediated ROS generation, and autophagy played a protective role against apoptosis through up-regulating the p38-NF-κB survival pathway in A375-S2 cells
He et al. (2014)	A	A375-S2	Physalin A induces iNOS expression and NO generation promoting apoptosis and autophagy in A375-S2 cells, however autophagy decreases NO production, reducing the rate of apoptosis and protecting cells from death
Ooi et al. (2013)	F	T-47D	Physalin F displayed cytotoxic effect (IC_50_ = 3.6 µg/ml) on human breast T-47D carcinoma by apoptosis through the activation of caspase-3 and c-myc pathways
Arai et al. (2014)	B, F, G, H, K, and isophysalin B	DU-145 and PANC1	Physalins B, F, H, and isophysalin B showed cytotoxic effect against tumor cells with aberrant Hedgehog signaling. Furthermore, only physalin H acts by inhibiting the Hedgehog pathway by inhibiting the formation of the GLI1-DNA complex
Ma et al. (2015)	B	HCT116	Physalin B displayed cytotoxic effect (IC_50_ = 1.35 µM) on human colon HCT116 cells through the induction of apoptosis from the inhibition of the ubiquitin/proteasome pathway mediated by the generation of mito-ROS and induction of incomplete autophagy. In addition, physalin B activated the MAP kinase pathway, which regulates autophagic and apoptotic responses
Kang et al. (2016)	A	A549	Physalin A inhibits the proliferation of A549 cells through the generation of ROS mediated by the p38 and ERK pathways that led to the expression of p53, p21, and cdc2 proteins and caused cell cycle arrest in the G2/M phase
Yang et al. (2016)	A, B, C, D, F, G, I, J, L, M, N, O, P, Z, isophysalin A, and six new physalins	HL60; SMMC-7721; A-549; MCF-7 and SW-480	Physalins B, F, and J presented the best profiles, with IC_50_ values above 5 µM to the cancer cells lines evaluated
Zhu et al. (2016)	A	H292; H1975; H358; H460 and A549	Physalin A showed antiproliferative effect in non-small cell lung cancer by activating apoptosis through inhibition of the JAK/STAT3 signaling pathway. In addition, physalin A significantly suppressed tumor xenograft growth
Sun et al. (2017a)	Physalins V, VI, VII, VIII, and IX	C4-2B; 22Rv1; 786-O; A-498; ACHN and A375-S2	Physalin B and F showed antiproliferative activities against all tested human cancer cells with IC_50_ values of 0.24–3.17 μM
Wang et al. (2018)	B	MCF-7; MDA-MB-231 and T-47D	Physalin B reduced the viability of MCF-7 cells by inducing wild-type p53 expression and inhibiting the Akt pathway. In addition, act in MDA-MB-231 and T47D cells by inactivating mutant p53, resulting in the induction of the arrest of the cell cycle in the G2/M phase and promoting the cleavage of PARP and caspases-3, -7, and -9 to initiate death by apoptosis
Chen et al. (2018)	F	SW480 and DLD-1	Physalin F inhibited the growth of SW40 and DLD-1 cells by inhibiting the Wnt glycoprotein and therefore promoted YAP-dependent β-catenin degradation. In addition, physalin F inhibited tumour growth by down-regulating β-catenin in tumour bearing mice
Boonsombat et al. (2020)	B, D, F, G, U, and XI	HL-60; MOLT-3; A549; HeLa; HuCCA-1; HepG2 and MDA-MB-231; T4D-7 and S102	Physalin B and F showed antiproliferative activities against all tested human cancer cells, with IC_50_ values of 0.38–29.71 μM
Cao et al. (2019)	B	A549	Physalin B downregulates the cyclin B1/CDK complex and causes cell cycle arrest in G2/M. It reduces mitochondrial ATP production, increases levels of reactive oxygen species, and elevates mitochondrial membrane potential, thereby inducing apoptosis of A549 cells
Shin et al. (2019)	A	Hepa-1c1c7 and HepG2	Physalin A reduces the cell viability of liver cancer cells by inducing Nrf2 expression via ERK and p38 pathways
Sun et al. (2021)	7b-ethoxyl-isophysalin C and 3b-ethoxyl-2,3-dihydro-4,7-didehydrophysalin B	PC-3	7b-ethoxyl-isophysalin C showed apparent moderate with IC_50_ values of 8.26 µM, whereas the other physalin exhibited no cytotoxicity against PC-3 cancer cell line
Fang et al. (2021)	B	HGC-27 and SGC-7901	Physalin B inhibited proliferation via cyclin-dependent kinase and induces caspase-dependent apoptosis in HGC-27 cells
Ko et al. (2021)	A	MDA-MB-231; MDA-MB-453; HCC-1937 and MCF-7	Physalin A inhibited proliferation and migration of breast cancer cells and mammospheres formation. In addition, physalin A inhibited the formation of breast cancer stem cells and decreased the transcript levels of BCSC-related genes (Oct4, CD44, Sox2, c-myc, and Nanog) via regulation of the Hedgehog/Hippo signaling pathway
Xu et al. (2021)	B, D, F, H, I, J, 5β, 6β-epoxyphysalin C, and 5α-chloro-6β-hydroxyphysalin C	PC-3; MCF-7; NCI-H460 and SF-268	Physalins F, H, 5β, 6β-epoxyphysalin C, and 5α-chloro-6β-hydroxyphysalin C presented selective cytotoxicity for at least one of the tested cancer cell lines
Yang et al. (2021)	B, D, and F	HT1080	Physalin F, through inhibition of isocitrate dehydrogenase enzyme, showed antiproliferative activity in HT1080 cell and induced apoptosis cells death

AKT, protein kinase B; ATP, adenosine triphosphate; BCSC, breast cancer stem cells; CDC2, cyclin-dependent kinase 1; EC_50_, effective concentration of 50%; ERK, extracellular signal-regulated kinases; GLI-1, glioma-associated oncogene; IC_50_, inhibitory concentration of 50%; iNOS, Nitric Oxide Synthases; JNK; c-Jun N-terminal kinase; MAP, microtubule-associated protein; NFκB, nuclear factor kappa-light-chain-enhancer of activated B cells; NO, nitric oxide; NRF2, nuclear factor erythroid 2-related factor 2; PARp, Poly (ADP-ribose) polymerase; ROS, reactive oxygen species; STAT3, Signal transducer and activator of transcription 3; TNF, tumor necrosis factor.

In general, the cytotoxic activity of physalins was shown to be related to induction of programmed cell death (Vandenberghe et al., 2008; Hsu et al., 2012; Wu et al., 2012; He et al., 2013a; Ma et al., 2015; Fang et al., 2021; Yang et al., 2021). Physalins A, B, and F may trigger apoptosis through activation of the intrinsic pathway of poly (ADP-ribose) polymerase (PARP) cleavage (Wu et al., 2012; He et al., 2013a; Ma et al., 2015). In this sense, Yang et al. (2021) also concluded that physalin F was able to induce apoptosis in HT1080 cells mainly through the inhibition of the enzyme isocitrate dehydrogenase (IDH). Conversely, Fang et al. (2021) demonstrated that, when physalin B treatment was performed in HGC-27 cells, the cell cycle-related proteins cyclin D1, cyclin D3, CDK4, CDK6, cyclin E, and the phosphorylated retinoblastoma tumor suppressor protein (p-Rb) were downregulated in a concentration-dependent manner, without activation of the intrinsic apoptosis pathway. Another important finding is that physalins stimulate the production of reactive oxygen species (ROS) and NO, which are important mediators responsible for triggering cell death by apoptosis (Wu et al., 2012; He et al., 2013b; He et al., 2014).

Physalin A was also shown to induce apoptosis, acting through p53-Noxa activation and ROS formation (He et al., 2013b). Kang et al. (2016) observed that p53-mediated production of ROS promoted cell cycle arrest in the G2/M phase in non-small cell lung cancer. The findings of Wang et al. (2018) indicate that physalin B induces cell death by apoptosis in a p53-dependent manner in breast cancer cells. In addition, physalin B causes cell cycle arrest in the G2/M phase, with an increase in p53 and p21 in cells of three breast cancer cell lines. Cell cycle arrest and increase in p53 and p21 were also described for physalin F in renal carcinoma cells (Wu et al., 2012). In contrast, physalin B has been shown to have an antiproliferative effect and apoptotic activity on A549 lung cancer cells regardless of increased p53 expression, but promoting the upregulation of p21 (Cao et al., 2019).

Physalin A was also shown to promote an increase in the expression of detoxifying enzymes through the activation of Nrf2 via ERK and p38 kinase, when tested in a HepG2 hepatocarcinoma model. This result suggests the suppression, in early stage of carcinogenesis, regulating the activity of phase II detoxification enzymes, indicating physalin A as a potential chemopreventive agent for liver cancer (Shin et al., 2019).

In contrast, Ma et al. (2015) showed that physalin B promotes activation of the ERK, JNK, and p38 pathways (MAPKs) through the stimulation of mito-ROS in human colorectal cancer cells (HCT116 strain), in a concentration and time dependent manner. Since this process was reversible with use of N-acetyl-*L*-cysteine (NAC), a ROS scavenger, it was suggested that the antitumor activity of physalin B is directly associated with the production of ROS (Ma et al., 2015). Corroborating with this data, Wu et al. (2012) showed that NAC could revert the apoptosis induced by physalin F in renal carcinoma cells (A498, ACHN, and UO-31).

**FIGURE 4:**
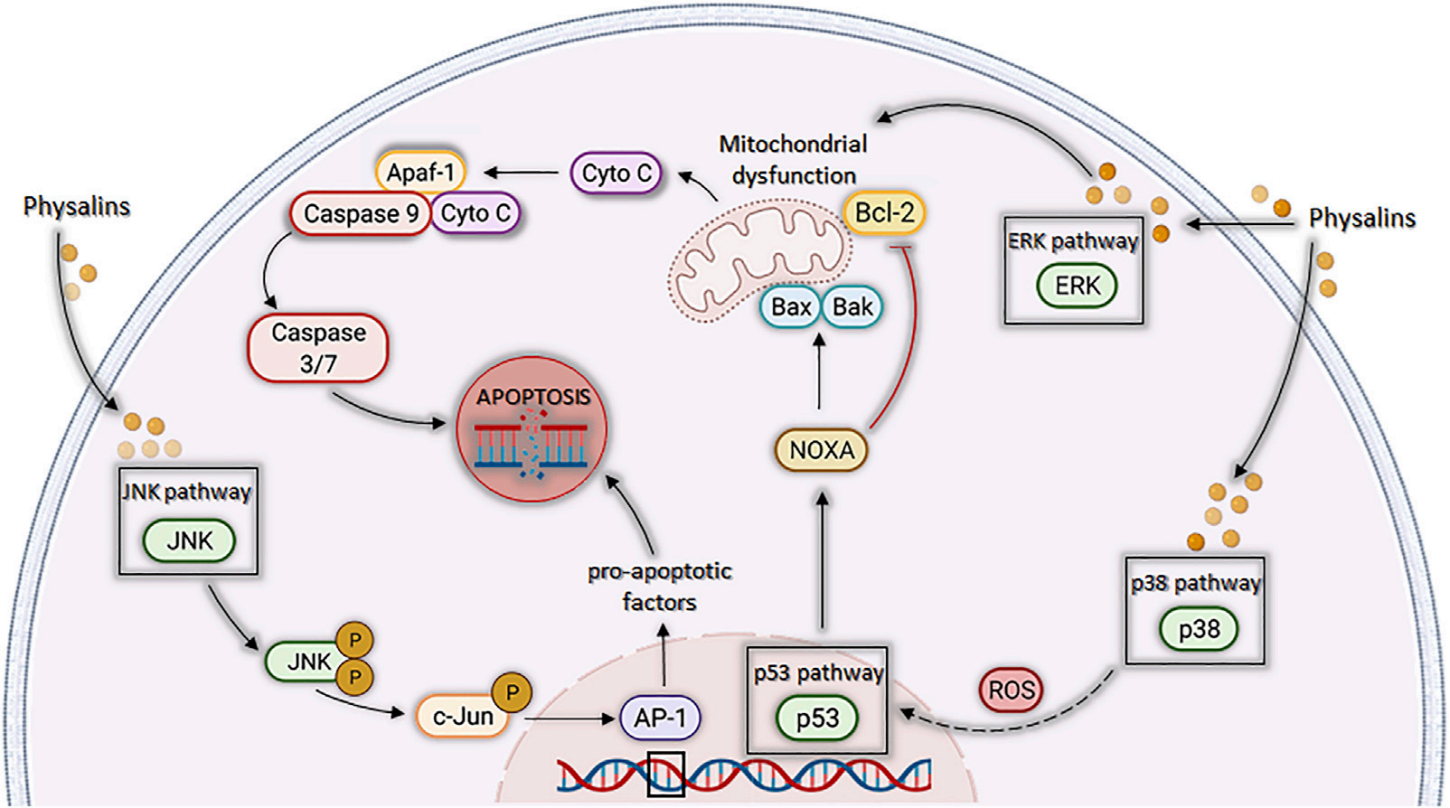
Cell death by apoptosis is induced by physalins through different pathways related to mitogen-activated protein kinase (MAPK). Physalins increase the phosphorylation levels of ERK1/2, JNK, and p38 MAPK. ERK1/2 activation induces mitochondrial ROS (mTOR) production, leading to the release of cytochrome c and activation of caspases 3, 6, and 9, triggering apoptosis. JNK activation promotes the phosphorylation of c-Jun, which leads to the formation of activator protein 1 (AP-1), a protein that regulates the transcription of pro-apoptotic factors and leads to apoptosis. P38 activation results in increase of ROS levels, which leads to p53 activation, which in turn increases the transcription of pro-apoptotic proteins, such as Noxa, BAX, and BAK, and decreases the transcription of the anti-apoptotic BCL-2 protein, leading to apoptosis through the mitochondrial pathway.

Additionally, physalins A and B caused a decrease in proliferation and viability of cancer cell lines by acting on MAPK pathways (Figure 4) (Han et al., 2011; Ma et al., 2015; Kang et al., 2016; Shin et al., 2019). Han et al. (2011) evaluated the activity of physalins A and B in prostate cancer cells (C42B and CWR22Rv1 cell lines), and the inhibition of cell proliferation correlated with activation of cell death mechanisms through ERK and JNK pathways. Corroborating the aforementioned findings, Kang et al. (2016) observed the growth inhibition of human lung carcinoma cells (A549 cell line) by physalin A, and this effect was associated with the activation of p38 and ERK pathways, the first being a pathway involved with the generation of ROS and the second linked to cell death by different mechanisms, mainly by the extrinsic apoptosis pathway (Cagnol and Chambard, 2010). Finally, Ma et al. (2015) observed an increase in the levels of ERK1/2, JNK, and p38 phosphorylation induced by physalin B, in a concentration and time dependent manner. Additionally, when inhibitors of these proteins were used, a partial reversion of PARP cleavage and p62 accumulation were seen, indicating that ERK, p38, and JNK pathways participated in both apoptosis and autophagy processes triggered by physalin B.

Another possible molecular target described for physalins is the ubiquitin/proteasome (UPP) pathway, which, together with the autophagy-lysosome pathway (ALP), is the main intracellular clearance system of eukaryotic proteins (Zhu et al., 2010). The inhibition of this pathway has been shown to induce apoptosis due to the cytotoxicity of accumulated ubiquitinated proteins (Crawford et al., 2011). Physalins B and C act as inhibitors of the ubiquitin/proteasome pathway, thus promoting apoptosis (Ausseil et al., 2007; Vandenberghe et al., 2008; Ma et al., 2015). Physalin F, in turn, was shown to increase the ubiquitinization of β-catenin and the proteasome pathway-dependent degradation in colorectal cancer cells, without inhibiting the ubiquitin/proteasome (Chen et al., 2018). According to Ma et al. (2015), physalin B acts as an indirect inhibitor of UPP, inducing the formation of autophagosomes in the cytoplasm, in addition to reducing the fusion between autophagosomes and lysosomes in HCT116 colon cancer cells. This suggested the induction of an incomplete autophagic response by physalin B, presenting structural changes in F-actin microtubules and microfilaments, inhibition of lysosomal degradation and, consequently, inhibition of the autophagic pathway.

In another study, physalin A promoted the induction of autophagy pathway, causing upregulation of p38-NF-κB, which antagonize with apoptosis cell death (Cheong et al., 2012; He et al., 2013b). Furthermore, physalin B induced the accumulation of LC3-II protein (important for the initiation of autophagosome formation), while Beclin 1 protein was reduced and no alteration in mTOR phosphorylation was seen, suggesting that Beclin1 and mTOR are not necessary for the autophagic response induced by physalin B (Ma et al., 2015). In contrast, He et al. (2013a) observed an important role of Beclin 1 in HT1080 cells (human fibrosarcoma) treated with physalin A, since this molecule was upregulated and led to conversion of LC3 I to LC3 II.

Therefore, both autophagy and UPP inhibition may lead to activation of apoptotic mechanisms, since both pathways eliminate toxic or harmful molecules and may lead to activation of cell death pathways when they are impaired (Yin et al., 2016; Zhao et al., 2016). Finally, it is noteworthy that autophagy plays a dual role in cancer cells. In some situations, it may have a cytoprotective effect, culminating in resistance to chemotherapy. In contrast, in other cases, cytotoxic effects were reported converging to autophagy-mediated cell death (Lefranc and Kiss, 2008; Silva et al., 2020).

**FIGURE 5:**
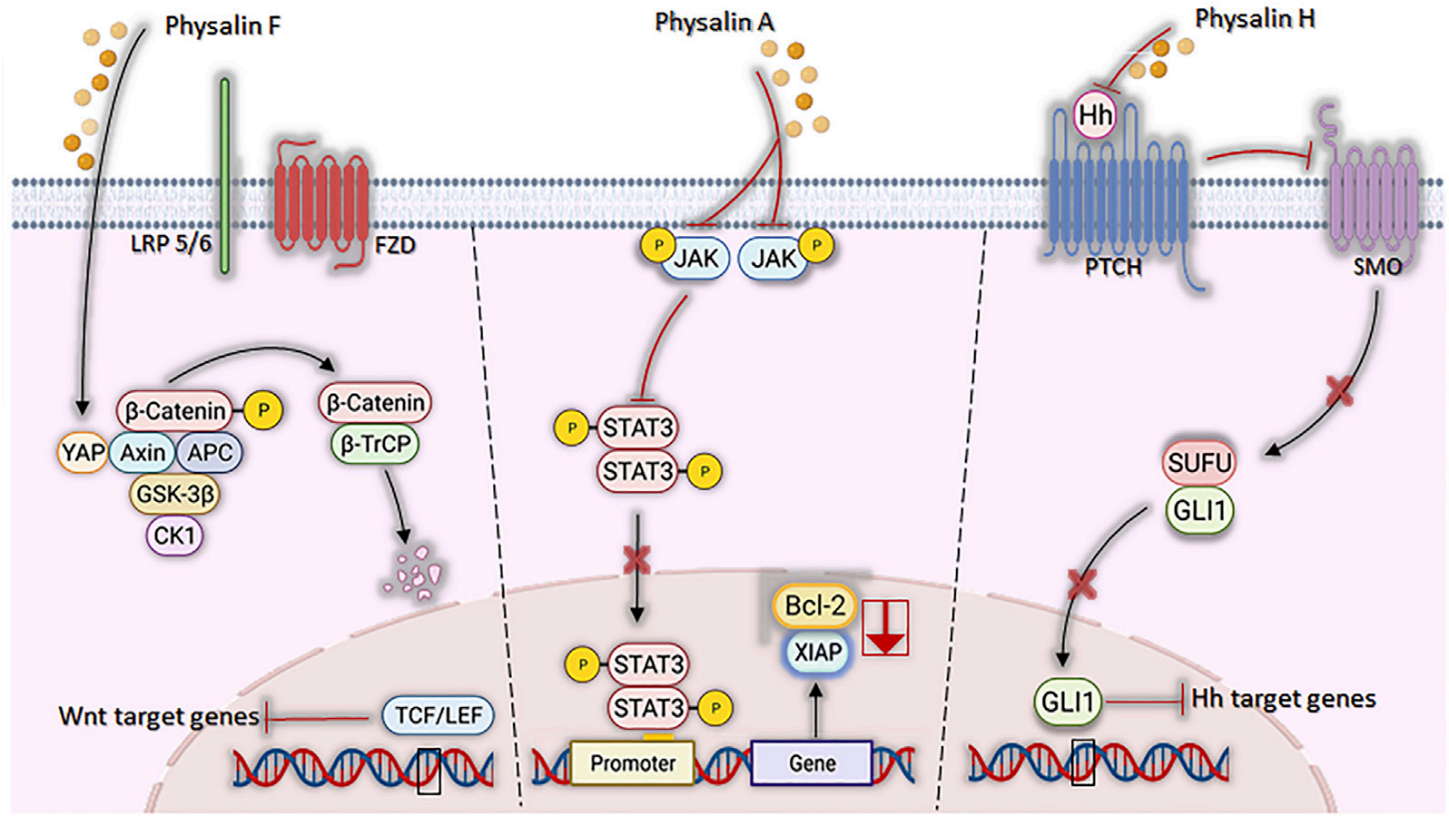
Mechanisms of action of physalins A, F, and H in aberrant signaling pathways. Physalin F inhibits Wnt/β-catenin signaling, accelerating the degradation of β-catenin and promoting the binding of YAP to the Axin, APC, CK1 and GSK-3β destruction complex. β-catenin phosphorylation facilitates its recognition by β-TrCP, leading to its degradation by the ubiquitin-dependent proteasome pathway. Physalin A inhibits the phosphorylation of the JAK receptor and the STAT3 protein, inhibiting their translocation to the nucleus and downstream Bcl-2 and XIAP transcription. Physalin H inhibits the Hedgehog pathway by suppressing Hh protein expression, impeding its binding to Hh-related proteins (PTCH) and inhibiting smoothened (SMO), which in turn allows the SUFU-containing GLI processing complex to generate transcriptional repressors, disrupting binding of GLI1 to its DNA binding domain and the non-expression of PTCH and Bcl-2.

Signaling pathways, crucial for the development and progression of some types of neoplasms under aberrant conditions, have their mechanisms attenuated by the actions of physalins (Figure 5). JAK/STAT3 pathway, suggested as a promising therapeutic strategy (Thomas et al., 2015), was inhibited by physalin A, both by suppressing JAK receptor phosphorylation and preventing STAT3 translocation to the nucleus and, consequently, inhibiting its transcriptional activity in non-small lung cell carcinoma (Zhu et al., 2016). STAT3, a transcription factor highly expressed and active in these cell lines, was less phosphorylated in Tyr705 in NCI-H1975 and U266 cells after physalin A treatment. Moreover, cell death by apoptosis was observed with a reduced expression of the anti-apoptotic genes Bcl2 and XIAP (Zhu et al., 2016).

Wingless-Int (Wnt) signaling dysfunction is associated with several types of cancer, such as colorectal cancer and the replication and maintenance of leukemic stem cells (White et al., 2012; Rodrigues et al., 2021). A study by Chen et al. (2018) showed that physalin F causes inhibition of Wnt glycoprotein binding to LRP5/6 and Frizzled receptors and promote β-catenin degradation through YAP (yes-associated protein) binding, when tested in colorectal cancer cells (Kim and Jho, 2014; Abylkassov and Xie, 2016).

Regarding the Hedgehog (Hh) signaling pathway, which acts on tissue homeostasis and embryonic development (Zhao et al., 2016), hedgehog (Hh)/GLI causes the formation and progression of a variety of neoplasms when in aberrant signaling, being also associated with the maintenance of cancer stem cells (Rodrigues et al., 2021). Physalins B and F are potent inhibitors of GLI-1 among PANC1 (pancreatic cancer) cells (Hosoya et al., 2008; Peukert and Miller-Mosilin, 2010), possibly by a mechanism associated with the inhibition of Hedgehog proteins, thus causing the interruption of the binding of GLIs to DNA (effector of Hedgehog signaling) (Jiang and Hui, 2008). Ko et al. (2021) found similar findings with physalin A in *in vitro* models of breast cancer, observing the inhibition of cancer cell proliferation/migration and mammosphere formation, associated with reduced expression of SMO and GLI1/2 proteins.

The NF-kβ pathway is also associated with the development and pathogenesis of cancer (Zhang et al., 2017; Xia et al., 2018; Rodrigues et al., 2021). Several studies have shown that physalins A, B, D, and F promote the inhibition of the NF-kβ pathway by different mechanisms, leading to apoptosis induction (Jacobo-Herrera et al., 2006; Vandenberghe et al., 2008; Wu et al., 2012; He et al., 2013b). In contrast, the work by Zhu et al. (2016) showed that physalin A did not affect the NF-kβ pathway in non-small cell lung carcinoma H292, H358, and H1975 cell lines.

Finally, physalins A and B interact with receptors that are overexpressed in some cancers, such as the androgen receptor (AR) (Han et al., 2011). In many cases, patients with the androgen-dependent form and who have already started chemotherapy develop the androgen-independent form and, therefore, are no longer responsive to treatment. In the independent form, although a constitutive expression of AR is found, it no longer responds to androgens (Kaarbø et al., 2007; Saraon et al., 2014). Han et al. (2011) showed that physalins A and B inhibit cell proliferation and reduce AR expression in C42B (androgen-dependent) and CWR22Rv1 (androgen-independent) lines, with C42B showing a stronger response than CWR22Rv1. In addition, low production of prostate-specific antigen (PSA) was observed in C42B cells after physalin treatment, a process regulated by the ERK and JNK pathways, which trigger cell death by apoptosis.

Regarding the *in vivo* antitumor activity, so far only physalins A, B, D, and F were investigated. All of these physalins reduced tumor growth, with the exception of physalin D, in a model of lymphocytic leukemia (Antoun et al., 1981; Chiang et al., 1992; Magalhães et al., 2010; Zhu et al., 2016; Chen et al., 2018). In addition, these physalins decreased the number of ki67-positive tumor cells, which is a well-known marker of cell proliferation (Magalhães et al., 2010; Zhu et al., 2016; Chen et al., 2018). In most cases, the antitumor effect of physalins was not accompanied by weight changes in the animals or signs of toxicity. The only exception was the toxic effects observed in the kidney of mice inoculated with sarcoma 180 tumor cells and treated with physalin B or D (Magalhães et al., 2010).

Despite the promising antitumor effect of physalins, their mechanism of action in animal models are poorly described. Physalin A suppressed tumor growth in a xenograft model using human NSCLC H292 (non-small cell lung cancer cell line), and its effects were related to an increase in caspase-3 activation and inhibition of JAK-STAT3 signaling (Zhu et al., 2016). In another xenograft model with the SW480 cell line (colon adenocarcinoma), physalin F suppressed tumor growth by down-regulating β-catenin in tumour-bearing mice (Chen et al., 2018). Although many studies have demonstrated the cytotoxic potential of physalins on several cell lines *in vitro* (Table 3), more *in vivo* experiments are still needed to ensure the safety and effectiveness of this class of compounds.

## Concluding Remarks and Future Perspectives

Physalins are versatile molecules that act in several cell signaling pathways and activate different mechanisms of cell death or immunomodulation. It is expected that new physalins can be purified, which can result in the discovery of more active physalins. In addition, chemical synthesis to obtain physalins needs to be better explored, since the purification of physalins from natural sources is a time-consuming, costly and not environmentally friendly process that results in a low yield. Due to the fast growth of the plants, which are annual herbs, an approach that has been investigated is the use of a *physalis* extract concentrated in the physalins, which has shown both low toxicity as well as pharmacological effects (Nogueira et al., 2013; Meira et al., 2015; Daltro et al., 2020; Do Espírito Santo et al., 2021).

Among the physalins evaluated, physalins B and F have the most potent effects, and therefore are the most promising physalins described so far. However, its mechanisms of actions, toxicological tests and *in vivo* activities need to be better characterized in further investigations to allow transposing the use of physalins in clinical studies. In conclusion, the physalin class is a promising source for the discovery of promising cytotoxic, immunomodulatory, and antiparasitic agents.
